# Wanderli Pedro Tadei (★1948 †2021)

**DOI:** 10.1590/0037-8682-0388-2021

**Published:** 2021-08-20

**Authors:** Vera Margarete Scarpassa, Joselita Maria Mendes dos Santos, Míriam Silva Rafael

**Affiliations:** 1 Instituto Nacional de Pesquisas da Amazônia, Laboratório de Genética de Populações e Evolução de Mosquitos Vetores, Coordenação de Biodiversidade, Manaus, AM, Brasil.; 2 Instituto Nacional de Pesquisas da Amazônia, Laboratório de Malária e Dengue, Coordenação de Sociedade, Ambiente e Saúde, Manaus, AM, Brasil.

**Figure:**
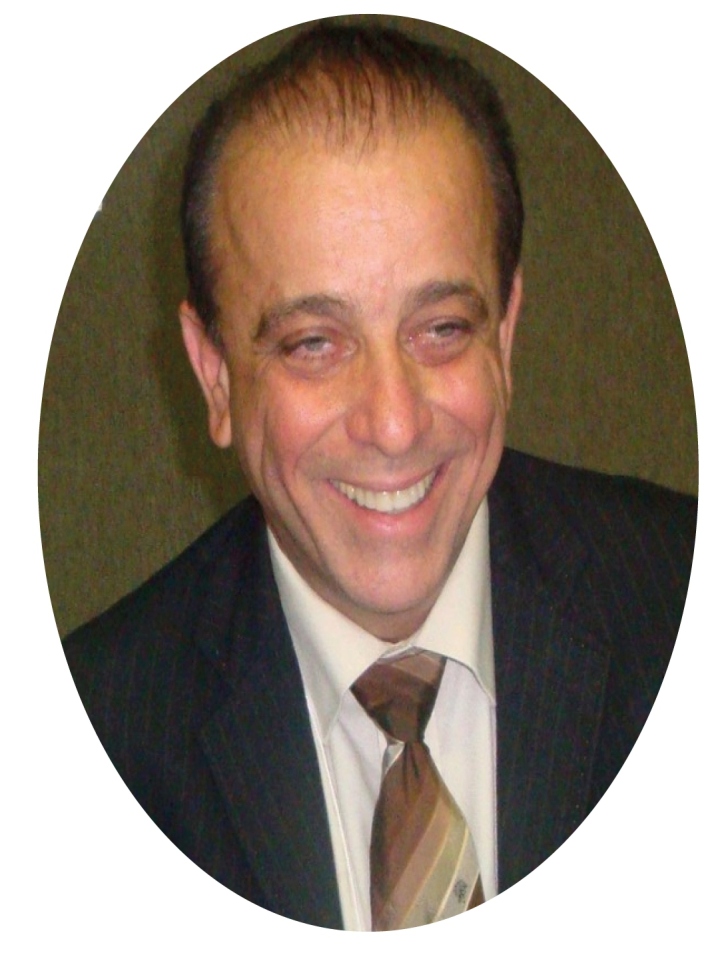

Senior Researcher and Professor Wanderli Pedro Tadei was born in the city of Urupês, State of São Paulo, on March 24, 1948, and he was the third son in a family of six. Professor Tadei studied in public elementary and middle schools. In 1971, he graduated in natural history by the Faculty of Philosophy, Sciences and Letters (currently Instituto de Biociências, Letras e Ciências Exatas-IBILCE), São Paulo State University (UNESP), São José do Rio Preto campus, State of São Paulo. He received his Master’s degree (1974) and doctoral (1977) degree in genetics from the University of São Paulo (USP), São Paulo city, wherein he investigated population cytogenetics, reproductive isolation, introgressive hybridization, and adaptedness (the state of be adapted) of *Drosophila prosaltans* and *Drosophila saltans*, and he received a fellowship from Fundação de Amparo à Pesquisa do Estado de São Paulo (FAPESP). In 1975, he passed a public examination and was admitted to the teaching staff of the UNESP Department of Biology, Presidente Prudente campus, State of São Paulo. In 1978, with the deactivation of the Biological Sciences course in that campus, he joined the UNESP Biology Department, São José do Rio Preto campus, where he taught embryology and evolution until July 1982. In 1978, Professor Tadei was invited by Dr. Warwick Kerr, the director of the Instituto Nacional de Pesquisas da Amazônia (INPA) at that time, to continue chromosome polymorphism studies of *Anopheles darlingi*, the main malaria vector in the Amazon region. This was a study previously initiated by Dr. Mohamed Rabbani, who died due to *Plasmodium falciparum* malaria. Professor Tadei accepted this challenge, and in January 1979, he traveled for the first time to the Amazon, a region that he loved at first sight. During the next two years, he traveled to the Amazon to collect *A. darlingi*, and polytene chromosome analyses were conducted at the UNESP. In July 1982, Professor Tadei moved permanently to the INPA in Manaus and remained there until March 2020, when he left to São José do Rio Preto for health reasons. On May 11, 2021, he died at 73 years of age. At the INPA, he worked very hard, made the Institute his second home, and made many friends. As Head of Laboratory of Malaria (currently Laboratory of Malaria and Dengue), Professor Tadei advanced genetic studies of *A. darlingi*. He also expanded field studies of biology, ecology, behavioral patterns of anophelines, malaria transmission dynamics, and surveillance and control methods of *Anopheles*, *Aedes*, and *Culex* using chemical and biological insecticides. In the 1980s, he led a project on malaria vectors in Rondônia, with a grant from the World Bank. At that time, Rondônia was the Brazilian state with the highest incidence of malaria. He also established partnerships with the Fundação Nacional de Saúde (FUNASA) and carried out training for healthcare personnel. With decentralization of the FUNASA to the states and municipalities, the Health Surveillance Agency of the Amazonas state was created, and partnerships between the INPA and this agency continued. During 1998-1999, he was invited by Peru’s Ministry of Health to discuss malaria control strategies in that country. Always smiling and polite, with charisma, which was his trademark, Professor Tadei had an extraordinary scientific leadership quality, leading or participating in large multidisciplinary projects, such as ELETRONORTE, FURNAS, PIATAM/CTPETRO/PETROBRAS, Usina Hidrelétrica Jirau/ESBR/ANEEL, and INCT-ADAPTA, among others. He also led projects that aimed at studying anophelines, with grants from the CNPq, FINEP, and FAPEAM, among others, and that collaborated with national and international scientists. 

Professor Tadei and his team conducted studies on mosquitoes in all states from the northern region and in the Maranhão state in the northeastern region of Brazil. He also participated in the *A. darlingi* genome project, where the INPA played a major role. In partnership with researchers and students, he developed not only the cravo-da-Índia solution to fight malaria vectors but also the lime and chlorine mixture used in house construction sites to kill larvae of the mosquito dengue vector. For ten years (2003-2013), he organized high-level vector biology courses, including theoretical and practical content, and lecture modules presented by renowned national and international experts. Professor Tadei published 200 scientific articles in national and international journals, 26 book chapters, two books, and a couple of patents. In addition, he has presented hundreds of congresses and scientific meeting abstracts of papers as well as hundreds of conferences and interviews. His articles have been widely cited worldwide. He was a faculty member in two postgraduate courses (Entomology and Genetics) at the INPA, biotechnology course at the University of State Amazonas, and biotechnology course and Bionorte network at the Federal University of Amazonas. He made major contributions to human resource training, having trained more than 75 masters and doctoral students, and more than 70 undergraduate students and post-doctoral fellows, many of whom are currently scientists and teachers in important Brazilian institutions. In 1999, he was designated as a member of the New York Academy of Sciences. In recognition of his extensive contributions to science and technology of the Amazon region, he received more than two dozen honors, including Medalha Ordem do Mérito do Poder Legislativo of State of Amazonas, Award Bahiense Ney Lacerda da Fundação de Vigilância e Saúde, Title of Amazonian Citizen, and Medalha Menção Honrosa Rio Negro, among others^1^. 

More recently, Professor Tadei has been honored by giving his name to a new species of Anophelines - *Anopheles tadei* ^2^. He also held administrative positions at the INPA: Head of Laboratory of Malaria and Dengue (1983-2020), Coordinator of Post-Graduation courses (1984-1986), Coordinator of Post-Graduation in Entomology (1987), Chief of Department of Health Science (1990-1997), Coordinator of Research (1998-2002), and Vice-Director (2006-2010). In addition to his research and teaching activities, he assiduously made contributions to charities, helped friends and persons in need, and worked on environmental issues.

We deeply regret the loss of this great scientist who made abundant contributions to the Brazilian Amazon region and to Brazil. He leaves a great legacy - extensive contribution to anopheline studies and other mosquitoes in the Brazilian Amazon region.

 Thank you, Professor and friend Tadei. The scientific community will miss you.
